# Wenyang Huazhuo Tuihuang Formula Inhibits the Th17/Treg Cell Imbalance and Protects against Acute-on-Chronic Liver Failure

**DOI:** 10.1155/2022/5652172

**Published:** 2022-03-30

**Authors:** Xiufeng Wang, Yunqing Zhong, Rongzhen Zhang, Yueqiao Chen, Minggang Wang, Chao Lv, Dewen Mao

**Affiliations:** ^1^Department of Liver Disease Area 1, The First Affiliated Hospital of Guangxi University of Chinese Medicine, Nanning 530201, China; ^2^Department of Pulmonary Disease, Guangxi International Zhuang Medicine Hospital, Nanning, China

## Abstract

**Objective:**

Acute-on-chronic liver failure (ACLF) is a group of chronic liver diseases and caused by acute internal and external liver injury. Wenyang Huazhuo Tuihuang (WYHZTH) formula had a good clinical effect on promoting the resolution of jaundice. The aim of this study is to further investigate the mechanism of the WYHZTH formula in the ACLF rat model.

**Methods:**

The ACLF rat model was constructed by combining human serum albumin with LPS and D-gal. WYHZTH was used to intervene and treat. The cytokines IL-17, IL-23, IL-10, and TGF-*β* were detected by ELISA and fluorescence-quantitative PCR. Flow cytometry was used to detect the percentage of Th17 and Treg cells in the peripheral blood and liver tissues of each group of rats. The pathological changes in the liver tissue were detected by hematoxylin-eosin staining, immunohistochemistry, and electron microscopy.

**Results:**

Compared with the ACLF group, the WYHZTH formula and Thy significantly decreased the levels of ALT, AST, and CHE in the ACLF group. After drug intervention, apoptosis was significantly reduced. The PCNA expression decreased in the ACLF model group but increased in the WYHZTH or Thy group. Under transmission electron microscope, hepatocytes in the ACLF group showed obvious necrosis. After drug intervention, hepatocyte necrosis was reduced with most of the structure returning to normal.

**Conclusion:**

This present study demonstrated that WYHZTH formula may protect against acute-on-chronic liver failure, which may be related to the inhibition of Th17/Treg cell imbalance.

## 1. Introduction

Liver failure is a serious clinical syndrome, which is characterized by massive necrosis of hepatocytes caused by various acute or chronic injuries caused by drinking, hepatotoxic drugs, or virus infection, such as hepatitis B virus (HBV) and hepatitis C virus (HCV) [1–3]. Acute-on-chronic liver failure (ACLF) is a concept that was first widely used in liver disease intensive care units to distinguish patients who were treated with artificial livers as a way to liver transplantation [4]. In 2009, the Asia Pacific Association for the Study of the Liver (APASL) first defined the concept of ACLF. ACLF is an acute injury of liver function that occurs on the basis of chronic liver disease, and it is related to multiple organ failure and high mortality [5–7]. Therefore, new strategies to prevent the disease progression of liver failure are urgently required.

In recent years, Th17/Treg balance in liver failure has become a research hotspot [8, 9]. CD4+ T lymphocytes, as helper T cells, play an important regulatory role in the body's immune response and inflammatory response, and Treg/Th17 is derived from the initial CD4+ T cells (Th0), which antagonize each other in differentiation and function, and is closely related to the inflammatory response, which is involved in the occurrence of viral hepatitis, cirrhosis, and acute or chronic liver failure [10–13].

Currently, the treatment of liver failure mostly adopts comprehensive measures such as removing the cause and regulating immune function, but there is still no breakthrough progress, and there are many complications and high mortality. So far, there was no western medicine that could inhibit the progress of ACLF, while in a previous study, Wenyang Huazhuo Tuihuang (WYHZTH) formula is composed of Baifu tablets, dried ginger, Yin Chen, raw rhubarb, Chishao, *Polygonum cuspidatum*, ginseng, atractylodes, and licorice, which have a good clinical effect on promoting the resolution of jaundice and removing the dampness of the liver, gallbladder, spleen, and stomach, thereby clearing away heat and removing fire. At the same time, studies have pointed out that it also has a diuretic effect [14, 15], which shows a delightful prospect for liver function [16].

Our study was to deeply explore the mechanism of WYHZTH formula in the ACLF rat model. We intended to use Wenyang Huazhuo Tuihuang Recipe (WYHZTHR) to treat the ACLF model, evaluate the expression changes of CD4+ T cell subsets, clarify the protective effect of WYHZTHR on liver tissue damage, explore the targets of action of Chinese medicine in the treatment of ACLF, and provide a new theoretical basis for the prevention and treatment of ACLF by Chinese medicine.

## 2. Materials and Methods

### 2.1. Materials

The herbal WYHZTH formula was decocted by the Traditional Chinese Medicine Laboratory Center of the First Affiliated Hospital of Guangxi University after the identification of crude drugs was confirmed. Thymopentin injection (M107827, Aladdin), 20% human serum albumin (HSA), D-galactosamine (D-Gal), and lipopolysaccharide (LPS) were purchased from Sigma. The other reagents used were as follows: hematoxylin-eosin (Cat. No. E8090, G1140, G8590, Solarbio); protein marker, BCA protein concentration determination kit (Cat. No. XY-MY-0112, XY-MY-0096, Shanghai Xuanya), PVDF membrane, ECL luminescence reagent (Cat. No. XF- P3360, ZDSJ140, Xinfan Company), Tween-20 (Cat. No. PW0028, LEAGEN Company); RIPA tissue cell rapid lysate (Cat. No. BL504A, Biosharp); Nephrin, Podocin, LC3-I/II, Beclin1, P62, AMPK, and GAPDH protein primary antibody (Cat. Nos. PAB40854, PAB44275, PAB34124, PAB44768, PAB35470, PAB30970, and PAB36269, Bioswamp); p-AMPK and p-ULK1 (Cat. Nos. 50081S and 14202S, CST); goat anti-rabbit IgG (Cat. No. SAB43714, Bioswamp); and MaxVision TM secondary antibody and HRP-polymer (Cat. No. Kit-5020, Maixin).

### 2.2. Animal Model

Sprague Dawley (SD) rats, equal number of rats of either sex, 8 weeks old, weighing about 250 g, with a total of 90 were used. The animals come from Three Gorges University. Laboratory animal license number: SYXK (E) 2018-0104, certificate No. 42010200003097. They were raised under SPF conditions. The temperature was 22–26°C, relative humidity 50–60%, artificial light and dark for 12 hours, and adaptive feeding for 1 week.

According to the random number table method, 15 rats were selected as the normal control group and the other 75 rats were model groups. 20% human serum albumin was used to construct a rat model of liver cirrhosis [17]. The rats were injected with 20% human serum albumin at 15 mg/kg through the tail vein twice a week, and the model was formed after 6 weeks. Based on liver cirrhosis, the ACLF rat model was constructed by intraperitoneal injection of lipopolysaccharide (LPS 100 *μ*g/kg) and D-galactosamine (D-Gal 400 mg/kg) [17].

### 2.3. Drug Intervention and Animal Groups

After the model was successfully constructed, the rats were divided into 6 groups for drug intervention, each with 15 rats: ①Normal control group (NC): no treatment, fed with ordinary feed; ②Acute-on-chronic liver failure model group (ACLF): equal volume of drinking water gavage. ③Thymopentin group (ACLF + Thy) : thymopentin 0.11 mg/(kg·d) injected and administered for 6 weeks; ④WYHZTH low-dose group (ACLF + WYHZTH-L): 0.23 g/(kg·d) was given by intragastric administration for 6 weeks; ⑤WYHZTH middle-dose group (ACLF + WYHZTH-M): 0.46 g/(kg·d) suspension was gavaged for 6 weeks; and ⑥WYHZTH high-dose group (ACLF + WYHZTH-H): 0.92 g/(kg·d) suspension was gavaged for 6 weeks. The WYHZTH group and the thymopentin group were planned to be administered by gavage 5 days before the model was started, continued until 48 h after the success of the model, twice a day with an interval of 12 h, and the amount of gavage liquid was 2 mL/100 g per day.

### 2.4. General Behavior Observation

During the experiment, the body weight (BW) of the rats was recorded every week, and the weight changes of the rats in each group compared with the NC group were statistically analyzed.

### 2.5. Biochemical Index Detection

After aseptic blood collection from the abdominal aorta, the whole blood was centrifuged at 3000 rpm for 15 min, and the serum was collected. The alanine aminotransferase (ALT), aspartate aminotransferase (AST), and cholinesterase (CHE) in the serum were detected by an automatic biochemical analyzer.

### 2.6. HE Staining

After the drug intervention completed, all rats were sacrificed. The liver tissue was dissected and then fixed with 4% paraformaldehyde solution for more than 24 hours, dehydrated with absolute ethanol, respectively, at 70%, 80%, 90%, and 95% gradient elution for 30 min, dehydrated with absolute ethanol twice and transparent with xylene, embedded in paraffin, and cut into tissue slices with a thickness of 5 *μ*m. After staining, the pathological changes in the liver tissue were observed, especially hepatocyte damage, and photos were taken under a light microscope.

### 2.7. TUNEL Method

According to the literature [18], after fixation, the liver tissue is paraffin-embedded and sectioned. The sections had undergone routine dewaxing and hydration. According to TUNEL test kit instructions, DAB was added into the sections for color rendering and restained with hematoxylin. 5 high-power lens fields were randomly selected; each lens count 100 hepatocyte nuclei in each field, and the percentage of apoptotic cells was calculated.

### 2.8. Immunohistochemical Assay

After antigen retrieval, paraffin sections were placed in 3% H_2_O_2_ and blocked for 10 minutes to eliminate endogenous peroxidase activity. And then, it was blocked and incubated with 10% goat serum for 30 min. Primary antibody (proliferating cell nuclear antigen, PCNA, 1 : 200 dilution) was added and incubated at 4°C in a humidified box overnight. Then, the secondary antibody (1 : 200) was added dropwise and incubated at 37°C for 30 min. The substrate DAB was added to develop color. When the color change of the section was observed, the staining solution was washed off with tap water immediately. Hematoxylin was counterstained for 3 min, differentiated with 1% hydrochloric acid and alcohol, and rinsed with tap water for 10 min. Gradient alcohol dehydration was performed. Xylene is transparent, and the film is mounted with a neutral gum. The regeneration of liver cells is observed, and pictures were captured under a microscope.

### 2.9. Transmission Electron Microscope

The liver specimens were fixed with 2.5% glutaraldehyde and 1% hungry acid, embedded in epoxy resin and sectioned after being dehydrated by ethanol and acetone, and then double stained with saturated uranyl acetate and lead citrate. Finally, we observed the change in the ultrastructure of the liver tissue under a transmission electron microscope.

### 2.10. Enzyme-Linked Immunosorbent Assay

The serum of rats was collected in each group, 50 *μ*L of different concentrations of standards and samples to be tested on the ELISA plate and 50 *μ*L PBS were added to the blank wells. 50 *μ*L of enzyme-labeled IL-17, IL-23, IL-10, or TGF-*β* antibody was added to each well, except for blank wells. After sealing the plate with a sealing film, incubate at 37°C for 30 min, carefully remove the sealing film, discard the liquid, and spin dry. 50 *μ*L of A developer and 50 *μ*L of B developer were added and develop the color at 37°C for 10 min in the dark. Finally, 50 *μ*L of stop solution was added to stop the reaction and the absorbance (OD value) of each well was measured at 450 nm wavelength. A standard curve was drawn with the concentrations of the standards as the abscissa and the OD values as the ordinate, and the concentration of IL-17, IL-23, IL-10, and TGF-*β* was calculated in the sample according to the OD value.

### 2.11. Real-Time Fluorescence Quantitative PCR

The total RNA of liver tissue was extracted by the Trizol method; reverse transcription was performed according to the TAKARA rapid cDNA first-strand generation kit; the generated cDNA was used as a template for fluorescence quantitative PCR amplification. Designing and synthesizing of each qRT-PCR primer: IL-17-F CCCTCAGACTACCTCAACCG, IL-17-R GCTCTCAGGCTCCCTCTTC; IL-23-F TGCTGCTCACGGTCACTT, IL-23-RGCTTTGTGGCATCCTGG; IL-10-FGGTTGTCGTCTCATTCTGAAAGA, IL-10-R GGTAGAGGACCCAAGTTCGTTAAGA; TGF-*β*-F ACCAACTATTGCTTCAGCTC, TGF-*β*-RCTTGCAGGAGCGCACGATCA; FOXP3-FCTGGGAAGATGGCATTGAC, FOXP3-RCACTCTCCACTCGCACAAA; GAPDH-FCCTTCCGTGTTCCTAC, GAPDH-RGACAACCTGTTCCTCA. All primers were synthesized by Wuhan Tianyi Huiyuan Company. PCR reaction conditions were as follows: predenaturation at 95°C for 3 min; 95°C for 5 s, 56°C for 10 s, 72°C for 25 s, total 40 cycles, and 72°C for 10 min. The Bio-Rad fluorescent quantitative PCR instrument was used to determine the Ct value. The Ct value is standardized with the Ct value of GAPDH, and the relative fold is calculated by 2^−△△Ct^, and the data are recorded and carried out by ABIprism7300SDS software.

### 2.12. Flow Cytometry

1 ml of anticoagulated whole blood sample was taken from each group of rats. After separating the cells, IL-17 and CD4 labeling were used and the frequency of Th cells was detected. Meanwhile, CD4, CD25, and Foxp3 labeling was used, and the frequency of Treg cells was detected on a flow cytometer.

### 2.13. Statistical Analysis

SPSS 21.0 was used for data analysis. The *t*-test for the comparison of the two sample means was statistically processed. The data were expressed as the $\bar{x}$ ± *s*, and the comparison of percentages was performed by the *χ*^2^ test; the comparison between multiple groups was performed by one-way ANOVA analysis, and *P* < 0.05 indicates the difference was significant.

## 3. Results

### 3.1. Effect of WYHZTH on the Body Weight of ACLF Rats

After the success of the acute-on-chronic liver failure model, the weight of rats in the ACLF group gradually decreased over time. After 4 weeks, the weight in the ACLF group was significantly lower than that in the NC group (*P* < 0.01). Compared with the ACLF group, the weights in the ACLF + WYHZTH-L/M/H group were all increased (*P* < 0.05), and the weight in the ACLF + WYHZTH-H group and the ACLF + Thy group was significantly improved, which was gradually increased with the increase of the WYHZTH dose (*P* < 0.05, Table 1).

### 3.2. The Level of Serum Liver Function Indexes in ACLF Rats after WYHZTH Treatment

**Figure 1:**
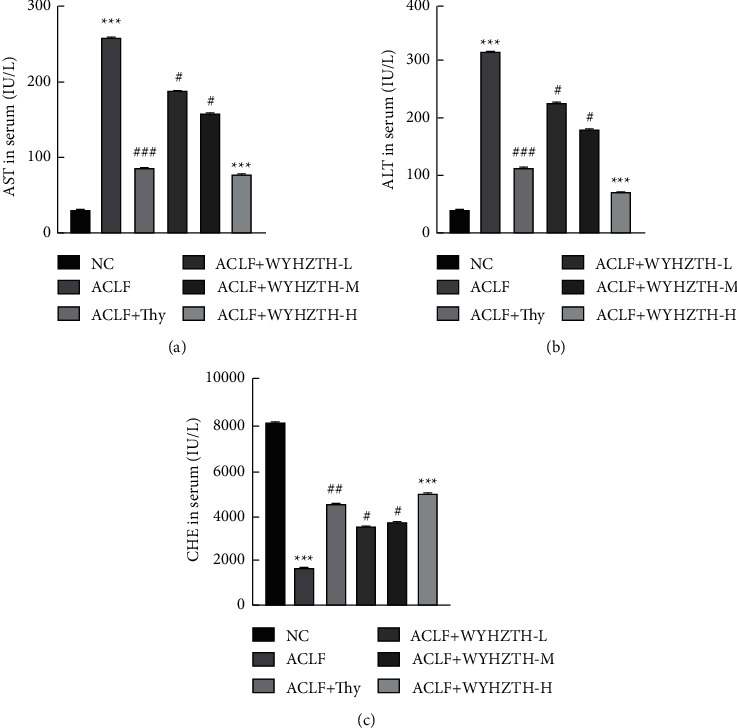
Effect of WYHZTH on the level of serum liver function indexes in ACLF rats. (a) The quantification of AST in serum of rats from different groups; (b) the concentration of ALT in serum of rats from different groups; (c) the concentration of CHE in serum of rats from different groups. vs. NC group, ^*∗*^*P* < 0.05, ^*∗∗*^*P* < 0.01, ^*∗∗∗*^*P* < 0.001; vs. DN group, ^#^*P* < 0.05, ^##^*P* < 0.01, ^###^*P* < 0.001.

In order to explore the effect of WYHZTH on the liver function of ACLF models, after the drug intervention was completed, we measured ALT, AST, and CHE in the serum. It was found that the levels of ALT and AST in the ACLF group were higher than those in the NC group (*P* < 0.001). Instead, the level of CHE was lower in the ACLF group. After treatment, the levels of ALT and AST in different doses of WYHZTH groups were significantly lower than those in the ACLF group (*P* < 0.05), but CHE was increased. Meanwhile, the content of ALT, AST, and CHE gradually changed with the WYHZTH dose increasing (Figure 1).

### 3.3. Effect of WYHZTH on the Morphology of Liver Tissue in ACLF Rats

**Figure 2:**
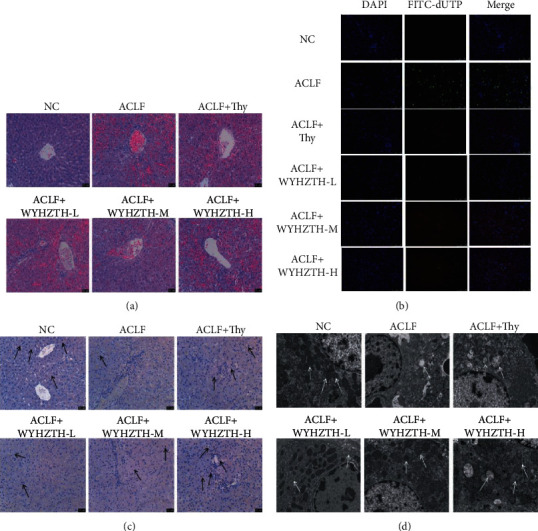
Effect of WYHZTH on the morphology of liver tissue in ACLF rats. (a) The liver tissue injury of rats in each group was observed by HE staining (200x); (b) the apoptosis change of liver tissue was tested by TUNEL staining (200x); (c) immunohistochemistry detected the expression of hepatocytes protein PCNA (200x); (d) ultrastructural changes of liver tissue in rats under electron microscope (6000x).

In order to further demonstrate the role of WYHZTH in the process of acute-on-chronic liver failure, the changes in the liver tissue structure of rats in each group were observed by HE staining (Figure 2(a)) and TUNEL staining for apoptosis change in the liver tissue (Figure 2(b)). Compared with the NC group, it was easy to find that the ACLF group showed ballooning and vacuolar degeneration of hepatocytes and the liver sinusoids dilated and congested in a small area, with a small amount of inflammatory cell infiltration outside the blood vessels and bile ducts. The degree of hyperemia and inflammatory cell infiltration in the ACLF + Thy group and the ACLF + WYHZTH group at different doses were significantly reduced (Figure 2(a)). It showed that the ACLF group significantly induced apoptosis in liver tissue in TUNEL staining. However, after drug intervention, there were some little green fluorescent spots; that is, apoptosis was significantly reduced (Figure 2(b)). The PCNA expression in the NC group was uniformly diffused, but expression decreased in the ACLF model group. In ACLF + Thy group and the different dose WYHZTH groups, PCNA increased in varying degrees. The arrows represented the local expression of PCNA (Figure 2(c)). Under transmission electron microscope, hepatocytes in the ACLF group showed obvious necrosis, mitochondria swelled, and cristae fractured or even disappeared, lipid droplets were seen in the cytoplasm, and lysosomes were significantly increased. After drug intervention, hepatocytes necrosis was reduced, and the degree of lesions was significantly reduced with most of the structure returning to normal. The arrows represented the distribution of mitochondrial cristae (Figure 2(d)).

### 3.4. Expression of Inflammatory Factors IL-17, IL-23, IL-10, and TGF-*β* in Serum and Liver Tissue of Rats

**Figure 3:**
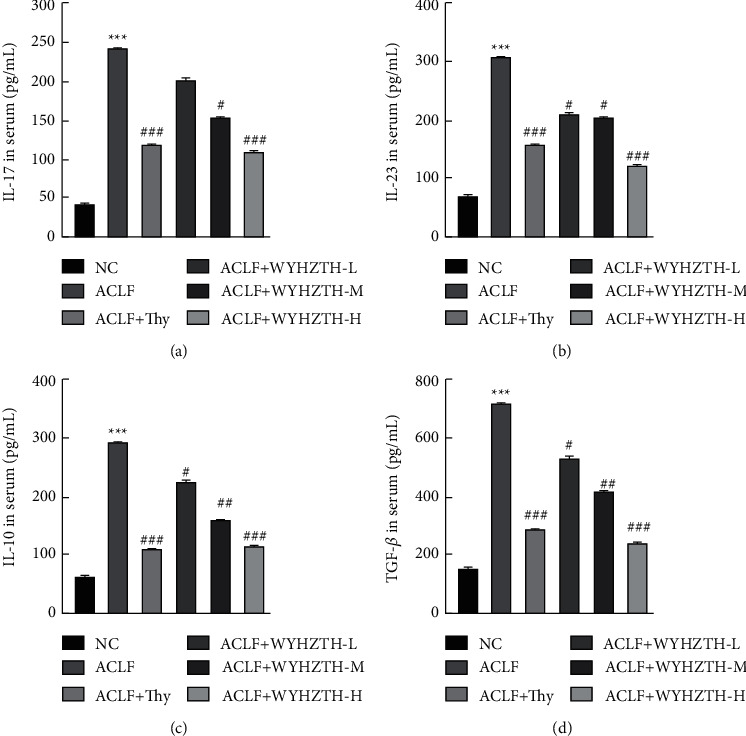
Expression of inflammatory factors IL-17, IL-23, IL-10, and TGF-*β* in serum of rats from different groups: (a) the concentration of IL-17 in serum of rats in each group; (b) the expression of IL-23 in serum from different groups; (c, d) the concentration of IL-10 and TGF-*β* in serum of rats from different groups. vs. NC group, ^*∗*^*P* < 0.05, ^*∗∗*^*P* < 0.01, ^*∗∗∗*^*P* < 0.001; vs. ACLF group, ^#^*P* < 0.05, ^##^*P* < 0.01, ^###^*P* < 0.001.

In order to clarify whether WYHZTH plays a role in improving the inflammatory injury of liver in ACLF, we first detected the expression of inflammatory factors IL-17, IL-23, IL-10, and TGF-*β* in the serum in each group of rats by ELISA. The results showed that the expressions of IL-17, IL-23, and IL-10 in the ACLF group were higher obviously than those in the NC group (*P* < 0.001); meantime, TGF-*β* was also increased in the ACLF group (*P* < 0.001, Figures 3(a)–3(d)). The levels of inflammatory factors in the ACLF + Thy and ACLF + WYHZTH-M/H groups were significantly lower than those in the ACLF group (*P* < 0.05, Figures 3(a)–3(d)). Moreover, as the dose of WYHZTH increasing, the expression of inflammatory factors and TGF-*β* gradually decreased. In the high-dose group, inflammatory factors and TGF-*β* were significantly reduced (*P* < 0.001, Figures 3(a)–3(d)), which was basically close to the normal level.

**Figure 4:**
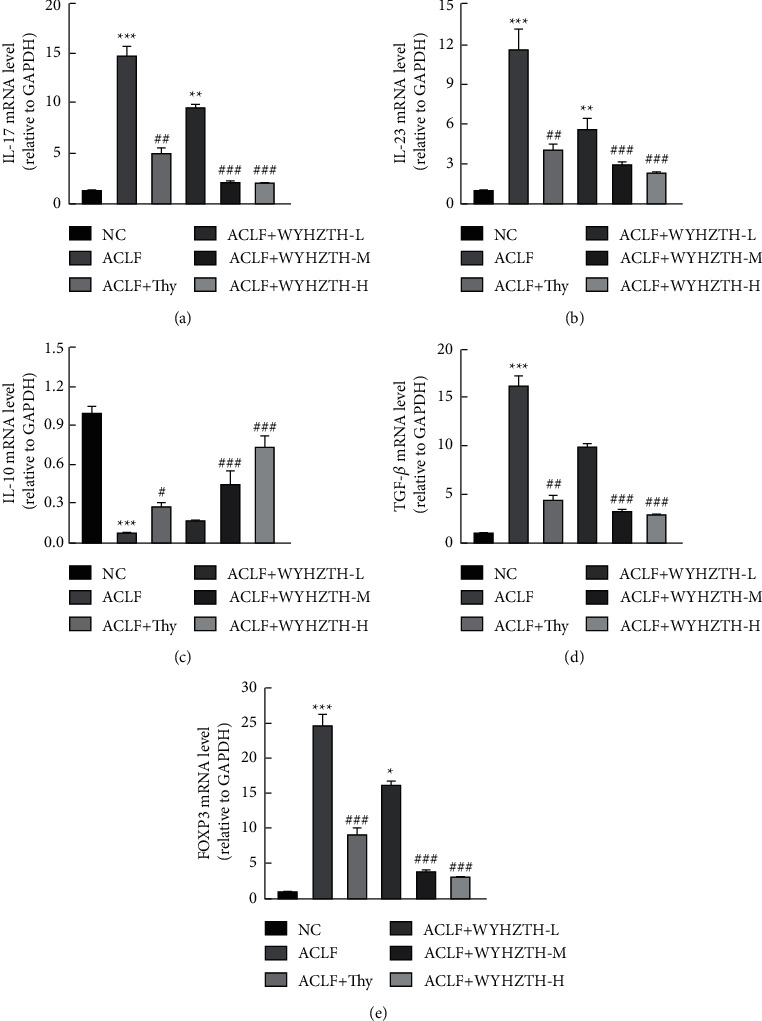
The mRNA level of IL-17, IL-23, IL-10, TGF-*β*, and FOXP3 in liver tissue of rats from different groups. (a) The mRNA level of IL-17 in liver tissue of rat in each group; (b) the mRNA level of IL-23 in liver tissue from different groups; (c–e) the mRNA level of IL-10, TGF-*β*, and FOXP3 in liver tissue of rats from different groups. vs. NC group, ^*∗*^*P* < 0.05, ^*∗∗*^*P* < 0.01, ^*∗∗∗*^*P* < 0.001; vs. ACLF group, ^#^*P* < 0.05, ^##^*P* < 0.01, ^###^*P* < 0.001.

In contrast to the cytokine level in the serum of rats, we further detected the mRNA levels of IL-17, IL-23, IL-10, TGF-*β*, and FOXP3 in liver tissues by fluorescence quantitative PCR. The results showed that compared to the NC group, the ACLF group IL-17, IL-23, TGF-*β*, and FOXP3 mRNA levels were significantly upregulated (all *P* < 0.05, Figure 4), but IL-10 mRNA was downregulated. After drug treatment, the expression of IL-17, IL-23, TGF-*β*, and FOXP3 was significantly reduced in ACLF + Thy and different doses of WYHZTH groups (*P* < 0.05, Figures 3(a)–3(d)). Meantime, the expression of inflammatory factors, TGF-*β* and FOXP3, all gradually changed with the dose of WYHZTH increasing.

### 3.5. The Frequency of Th17 and Treg Cells in the Peripheral Blood of Rats with ACLF and/or WYHZTH Intervention

**Figure 5:**
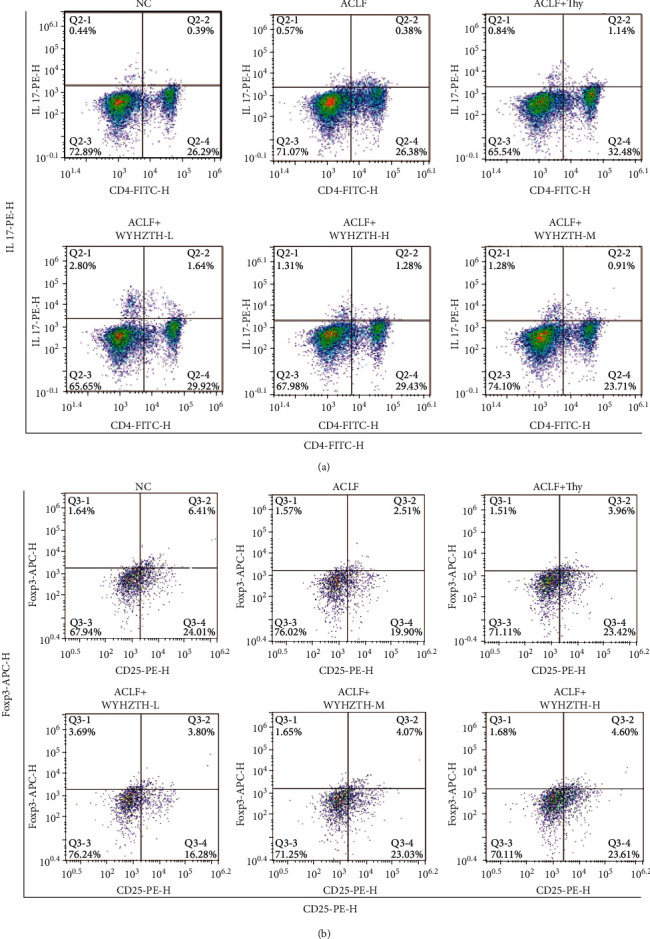
The frequency of Th17 and treg cells in the peripheral blood of rats with ACLF and/or WYHZTH intervention. (a) Flow cytometry detected IL-17 and CD4 labeled Th17 cells; (b) flow cytometry detected the frequency of CD25+Foxp3+ treg cells.

Studies have shown that the number of Th17 cells and Treg cells is closely related to the occurrence and progression of ACLF, so we further tested the frequency of Th17 and Treg cells in the peripheral blood of rats with ACLF and/or WYHZTH intervention to clarify the mechanism, by which WYHZTH can alleviate the occurrence and development of ACLF. Our results showed that the ratio of CD4+IL-17+ Th cells in the ACLF group was significantly higher than that in the NC group (Figure 5(a)), but after WYHZTH intervention, the ratio of CD4+IL-17+ Th cells decreased (Figure 5(a)), which gradually decreased with increasing dose. However, the opposite was true for CD4+CD25+Foxp3+ Treg cells. The ratio of Treg cells in the ACLF group was significantly lower than that in the NC group (Figure 5(b)), but after WYHZTH intervention, Treg cells gradually increased (Figure 5(b)), which was also dose-dependent with WYHZTH.

## 4. Discussion

Acute-on-chronic liver injury manifested as jaundice and coagulopathy [19, 20]. In APASL revised in 2014, the concept has been improved and high 28-day mortality rate has been added [21]. It is usually related to sudden trigger events, and the 90-day mortality rate increased due to multisystem organ failure [22, 23]. Acute-on-Chronic Liver Failure in Cirrhosis (CANONIC) is a large-scale prospective study of patients with decompensated acute liver cirrhosis. A multicenter study [24] aims to distinguish patients with high short-term mortality risk among people with decompensated cirrhosis and develops the definition of ACLF.

Among the earlier published studies included alcohol or bacteria-induced liver cirrhosis, decompensated liver cirrhosis, and ACLF [25]. This study found that systemic inflammation already exists when liver cirrhosis is decompensated, manifested by increased plasma inflammatory factors, renin and copeptin. These indicators are higher in ACLF patients. IL-6, IL-8, TNF-a, IL-10, and IL-1 are different in bacteria-induced ACLF and alcohol-induced ACLF. The severity of systemic inflammation is closely related to the frequency and severity of ACLF. The latter study included ACLF patients related to chronic hepatitis B in China [26]. The difference from the previous study is that it is not limited to the analysis of various factors in plasma but extends the scope of the study to immune cells. This study found that ACLF patients had higher peripheral blood white blood cell counts than those without ACLF. The neutrophil-to-lymphocyte ratio (NLR) is related to the death of severe hepatitis B patients. NLR can independently predict the occurrence and short-term mortality of ACLF.

So far, the Th17/Treg balance in liver failure has become a research hotspot. CD4+ T lymphocytes, as helper T cells, play an important regulatory role in the body's immune system and inflammatory response, and Treg/Th17 is derived from the initial CD4+ T cells (Th0), which antagonize each other in differentiation and function, and is closely related. Treg cells mostly secrete IL-10, TGF-*β*, and other cytokines to exert their inhibitory function, and Th17 cells mostly secrete IL-17, IL-23, TNF-*α*, etc. to exert their proinflammatory effects. Under normal physiological conditions, the body is in a balanced state. Once its steady state changes, it will affect the outcome of immune and inflammatory responses, which is closely related to the occurrence and progression of some immune diseases and inflammatory diseases. Th17 and Treg cells are derived from the same naive T cells [27, 28]. Under normal circumstances, the two maintain a balance, which is beneficial to the maintenance of the body's immune system in a stable state. Th17/Treg imbalance leads to excessive inflammatory response, which is involved in the occurrence of hepatitis, cirrhosis, and liver failure [26]. Liu et al. [29] compared the Th17/Treg ratio in patients with chronic hepatitis B with different disease progression levels in healthy people and a series of correlation analyses and confirmed that Th17/Treg is common in chronic HBV infection. The Th17/Treg ratio can more accurately reflect the progress of HBV infection-related liver disease. Shi Wenjuan [30] used ELISA to detect cytokines in the serum of 33 patients with ACHBLF and found that cytokines are related to the pathogenesis of ACHBLF with high levels of IL-17 and IL-35. The expression may be related to the occurrence of chronic hepatitis plus acute liver failure. Kan et al. [31] believed that ACHBLF patients have varying degrees of immune dysfunction.Th17/Treg imbalance is involved in the occurrence of hepatocyte inflammatory necrosis and can reflect the degree of liver inflammatory response. The above studies show that Treg cells, Th17 cells, and Treg/Th17 ratio are closely related to the progression of ACLF, and their role in the pathogenesis of ACLF is not very clear. Therefore, further in-depth research will provide more in-depth research on the pathogenesis and treatment of liver failure. Scientific theoretical basis.

Recently, the role of Th17/Treg balance in liver failure has become a hotspot. Treg cells mainly secrete IL-10, TGF-*β*, and other cytokines to exert their inhibitory function, but Th17 cells mostly secrete IL-17, IL-23, TNF-*α*, and other cytokines to exert their proinflammatory effects [32–34]. So we further tested the frequency of Th17 and Treg cells in the rats with ACLF and/or WYHZTH intervention, by which WYHZTH can improve the balance of Th17/Treg cells so as to maintain the stable secretion of inflammatory factors and alleviate the happening of ACLF.

Many clinical studies have reported on the advantages of traditional Chinese medicine in the treatment of liver failure, believing that it will enhance immune function and antiviral efficacy, thereby improving life quality. Traditional Chinese medicine does not have the name of “liver failure.” According to the symptom, it can be treated from “jaundice,” “paste yellow,” “abrupt yellow,” “heavenly yellow,” and other diseases. Although ACLF is caused by an epidemic virus when you feel it, it is due to the lack of righteousness in the body, and the epidemic virus can also cause damp heat, stagnation of blood, and loss of righteousness. With the in-depth study of liver failure, it is not difficult to find that spleen-yang deficiency and kidney-yang deficiency are more obvious in liver failure. Therefore, the method of invigorating the yang has become one of the common methods for the treatment of ACLF. The Center of Liver Diseases in the Guangxi University believes that the pathogenesis of ACLF can be summarized as poisonous turbidity and injury based on the theory of the Fuyang school of “yang governs yin from view” and liver failure “toxin-poisonous disease.” In the liver, qi deficiency and weakness, blood flow is not smooth; long-term qi deficiency will damage Yang. Combined with the research results of the research group's previous retrospective investigations, it is suggested that ACLF has certain characteristics of TCM syndrome distribution and evolution. Prolonged illness can easily lead to a loss of yang in the body. Yang deficiency and blood stasis yellow syndrome is the main type of ACLF [35]. According to this, the corresponding treatment methods should be to nourish yang, cultivate soil, detoxify, remove blood stasis, and retreat yellow, and condense the prescription of warming yang, dissolving turbidity, and retreating yellow. This prescription is an effective compound based on the treatment of acute liver failure with Jiedu Huayu Granules. Long-term clinical practice has shown that this prescription has good clinical effects in promoting the resolution of jaundice and reducing the mortality of liver failure. The whole prescription consists of Baifu tablets, dried ginger, atractylodes macrocephala, Yinchen, raw rhubarb, red peony root, knotweed, ginseng, and licorice. The combination of all the medicines has the effect of warming the sun to transform the turbidity, promoting blood circulation and relieving jaundice. The addition of Wenyang Huazhuo Tuihuang Decoction can reduce the proinflammatory factor IL-32 level and increase the inflammation inhibitory factor IL-10 level in patients with HBV-related ACLF, which has a certain regulatory effect on immune disorders [36]. But the specific mechanism of action is currently unclear.

## 5. Conclusion

In summary, our study established a rat ACLF model and found that WYHZTH can protect against liver failure by improving Th17/Treg balance, reducing liver damage, and playing a beneficial role in the ACLF rats. The mechanism of ACLF liver injury was elucidated from the perspective of congenital immunity, and the targets of action of Chinese medicine were explored in the treatment of ACLF, which will provide an effective target for the treatment and prognosis of ACLF. On the other hand, that also laid the foundation for our next research into the specific mechanism of WYHZTH and its relationship with innate immunity.

## Figures and Tables

**Table 1:** The effect of WYHZTH on body weight of rats with acute-on-chronic liver failure ($\bar{x}$ ± *s*, *n* = 6).

Group	BW (g)		
	After drug intervention		
	0 week	4 weeks	8 weeks
NC	266.37 ± 6.28	407.53 ± 4.21	478.53 ± 5.38
ACLF	259.60 ± 4.53	373.62 ± 6.42^*∗∗*^	419.30 ± 5.46^*∗∗∗*^
ACLF + Thy	260.28 ± 2.72	398.53 ± 6.23^##^	462.83 ± 6.34^###^
ACLF + WYHZTH-L	267.12 ± 2.64	368.53 ± 5.65	416.35 ± 2.35
ACLF + WYHZTH-M	265.42 ± 5.77	383.29 ± 1.59^#^	440.86 ± 6.71^##^
ACLF + WYHZTH-H	269.53 ± 3.26	401.78 ± 3.65^##^	466.54 ± 3.87^###^

vs. NC group, ^*∗*^*P* < 0.05, ^*∗∗*^*P* < 0.01, ^*∗∗∗*^*P* < 0.001; vs. ACLF group; ^#^*P* < 0.05, ^##^*P* < 0.01, ^###^*P* < 0.001.

## Data Availability

The data used to support the findings of this study are available from the corresponding author upon request.

## References

[1] Bernuau J., Rueff B., Benhamou J.-P. (1986). Fulminant and subfulminant liver failure: definitions and causes. *Seminars in Liver Disease*.

[2] Farci P., Alter H. J., Shimoda A. (1996). Hepatitis C virus-associated fulminant hepatic failure. *New England Journal of Medicine*.

[3] Navarro V. J., Senior J. R. (2006). Drug-related hepatotoxicity. *New England Journal of Medicine*.

[4] Kjaergard L. L., Liu J., Als-Nielsen B., Gluud C. (2003). Artificial and bioartificial support systems for acute and acute-on-chronic liver failure. *JAMA*.

[5] Zaccherini G., Weiss E., Moreau R. (2020). Acute-on-chronic liver failure: definitions, pathophysiology and principles of treatment. *JHEP Reports*.

[6] Li Q., Wang J., Lu M., Qiu Y., Lu H. (2020). Acute-on-chronic liver failure from chronic-hepatitis-B, who is the behind scenes. *Frontiers in Microbiology*.

[7] Yu Z., Zhang Y., Cao Y. (2021). A dynamic prediction model for prognosis of acute-on-chronic liver failure based on the trend of clinical indicators. *Scientific Reports*.

[8] Yu S. J., Jiang R., Mazzu Y. Z. (2016). Epigallocatechin-3-gallate prevents triptolide-induced hepatic injury by restoring the Th17/treg balance in mice. *The American Journal of Chinese Medicine*.

[9] Liang X. S., Li C. Z., Ying Z., Wan M.-B. (2012). Changes of Treg and Th17 cells balance in the development of acute and chronic hepatitis B virus infection. *BMC Gastroenterology*.

[10] Aleem A. A., Rahman E., Elgonimy A. (2011). Evaluation of CD4+CD25+ regulatory T cells in patients with hepatocellular carcinoma and liver cirrhosis. *Egyptian Journal of Hospital Medicine*.

[11] Lian J.-Q., Wang X.-Q., Zhang Y., Huang C.-X., Bai X.-F. (2009). Correlation of circulating TLR2/4 expression with CD3+/4+/8+ T cells and treg cells in HBV-related liver cirrhosis. *Viral Immunology*.

[12] Nasser S., Paul K. (2007). CD4+ T cell responses in hepatitis C virus infection. *World Journal of Gastroenterology*.

[13] Tang R., Lei Z., Wang X. (2020). Hepatitis B envelope antigen increases Tregs by converting CD4+CD25 T cells into CD4^+^CD25^+^Foxp^3+^tregs. *Experimental and Therapeutic Medicine*.

[14] Li X. Y., Meng C. Z., Ye B. Y. (2019). Qiao Chengli’s experiences in the treatment of nephritic syndrome with wenyang huazhuo therapy. *World Journal of Integrated Traditional and Western Medicine*.

[15] Zhang L. F., Wang Q., Yang L., Liu N., Shan Y., Bian H. (2018). Effect of Wenyang Huazhuo Tongluo decoction on VEGF, CTGF and ET-1 levels in systemic sclerosis mice. *Science Technology and Engineering*.

[16] An P., Dong S., Li X. F. (2020). Wenyang Huazhuo Fang exerts transient receptor potential cation channel subfamily C member-dependent nephroprotection in a rat model of doxorubicin-induced nephropathy. *Journal of Traditional Chinese Medicine: English Edition*.

[17] Liu X. H., Chen Y., Zhang J. (2007). The construction of acute-on-chronic liver failure rat model and its pathological mechanism explore. *Guide Paper of Technology*.

[18] Feldstein A. E., Canbay A., Angulo P. (2003). Hepatocyte apoptosis and fas expression are prominent features of human nonalcoholic steatohepatitis. *Gastroenterology*.

[19] Rao S., Ashraf I., Mir F., Samiullah S., Ibdah J., Tahan V. (2016). Dual infection with hepatitis B and epstein-barr virus presenting with severe jaundice, coagulopathy and hepatitis B virus chronicity outcome: 1996. *Official Journal of the American College of Gastroenterology ACG*.

[20] Sarin S. K., Choudhury A., Sharma M. K. (2019). Acute-on-chronic liver failure: consensus recommendations of the Asian Pacific association for the study of the liver (APASL): an update. *Hepatology International*.

[21] Sarin S., Kedarisetty C., Abbas Z. (2014). Acute-on-chronic liver failure: consensus recommendations of the Asian pacific association for the study of the liver (APASL). *Hepatology International*.

[22] Jalan R., Gines P., Olson J. C. (2012). Acute-on chronic liver failure. *Journal of Hepatology*.

[23] Olson J. C., Kamath P. S. (2011). Acute-on-chronic liver failure: concept, natural history, and prognosis. *Current Opinion in Critical Care*.

[24] Moreau R., Jalan R., Pere G. (2013). Acute-on-chronic liver failure is a distinct syndrome that develops in patients with acute decompensation of cirrhosis. *Gastroenterology*.

[25] Clària J., Stauber R. E., Coenraad M. J. (2016). Systemic inflammation in decompensated cirrhosis: characterization and role in acute-on-chronic liver failure. *Hepatology*.

[26] Wu W., Yan H., Zhao H. (2017). Characteristics of systemic inflammation in hepatitis B-precipitated ACLF: differentiate it from no-ACLF. *Liver International*.

[27] Edwards E. S. J., Bosco J. J., Aui P. M. (2019). Predominantly antibody-deficient patients with non-infectious complications have reduced naive B, treg, Th17, and Tfh17 cells. *Frontiers in Immunology*.

[28] Zhong X., Gao W., Degauque N. (2010). Reciprocal generation of Th1/Th17 and treg cells by B1 and B2 B cells. *European Journal of Immunology*.

[29] Liu H., You J., Chen H. (2016). The role of helper T lymphocyte 17 regulatory T lymphocyte balance in the progression of hepatitis B virus infection-related liver disease. *Chinese General Practice*.

[30] Shi W., Jia J., Li C., Wang H. (2016). The expression and significance of Th17/treg cytokines in patients with chronic hepatitis B acute liver failure. *Liver*.

[31] Kan Y., Gan J., Sun W., Feng T. (2016). Changes of Th17 and treg in patients with HBV-related chronic acute liver failure and their clinical relevance. *Liver*.

[32] Li C., Yang H., Rao X., Qiu X., Dou J. (2017). The research progress of Th17 cells and treg cells in regulating tumor immunity. *Pharmaceutical Biotechnology*.

[33] Bhattacharya P., Ghosh S., Ejazi S. A. (2016). Induction of IL-10 and TGF*β* from CD4+CD25+FoxP3+ T cells correlates with parasite load in Indian Kala-Azar patients infected with leishmania donovani. *PLoS Neglected Tropical Diseases*.

[34] Mills K. H. G., Allen A., Edwards S. (2014). S-16. *Cytokine*.

[35] Mao D., Wang N., Tang N., Long F., Chen X. (2015). Investigation on the constitution of traditional Chinese medicine in 232 patients with liver failure in Guangxi. *Journal of Integrated Traditional Chinese and Western Medicine Liver Disease*.

[36] Wang X., Zhao L., Zhong Y., Zhang R., Mao D. (2019). Effect of Wenyang Huazhuo Tuihuang decoction on serum IL-32, IL-10 and T cell subsets in patients with HBV-related ACLF. *Guangxi Traditional Chinese Medicine*.

